# Establishment of patient‐derived xenograft model of peritoneal mucinous carcinomatosis with signet ring cells and in vivo study on the efficacy and toxicity of intraperitoneal injection of 5‐fluorouracil

**DOI:** 10.1002/cam4.2766

**Published:** 2019-12-08

**Authors:** Yu‐Lin Lin, Jue Zhang, Feng‐Cai Yan, Xi Jiang, Ru Ma, Zhi‐Ran Yang, Hong‐Bin Xu, Zheng Peng, Qian Chen, Yan Li

**Affiliations:** ^1^ Department of Peritoneal Cancer Surgery Beijing Shijitan Hospital Capital Medical University Beijing China; ^2^ Department of Pathology Beijing Shijitan Hospital Capital Medical University Beijing China; ^3^ Department of Myxoma Aerospace Central Hospital Beijing China; ^4^ Department of General Surgery Chinese PLA General Hospital Beijing China; ^5^ Thorgene Co., Ltd. Beijing China

**Keywords:** 5‐FU, intraperitoneal injection, organ toxicity, PDX model, pseudomyxoma peritonei

## Abstract

**Background:**

Pseudomyxoma peritonei (PMP) is an indolent malignancy and insensitive to systemic chemotherapy. The authors established patient‐derived xenograft (PDX) model of PMP, and evaluated the efficacy and toxicity of intraperitoneal (i.p.) administration of 5‐fluorouracil (5‐FU) in this model.

**Methods:**

Human PMP sample was collected to establish subcutaneous (s.c.) and i.p. model. In vivo study of i.p. injection of 5‐FU was performed in i.p. model, with experimental peritoneal cancer index (ePCI) score and pathological examinations for evaluating the efficacy and toxicity.

**Results:**

Both s.c. and i.p. models were constructed. The average passage interval of s.c. model was 44.2 ± 5.2 days, and the i.p. model was characterized by disseminated solid tumor nodules in abdominal‐pelvic cavity. Both models were diagnosed as peritoneal mucinous carcinomatosis with signet ring cells (PMCA‐S). Immunohistochemical characteristics was similar to human. *GNAS* mutation was detected in both model and patient. In the in vivo study, average ePCI of treatment group was lower than control and vehicle group (*P* = .004). Histopathology revealed obvious tumor necrosis in treatment group, and decreased Ki67 positive rate (*P* = .010). In toxicity study, 5‐FU significantly influenced body weight (*P* = .010) and 1 animal from treatment group died on day 14. Congestive splenomegaly was observed (88.9%). Hepatotoxicity presented as acidophilic body (55.6%), cholestasis (100%), bile canaliculus hyperplasia and obstruction (22.2%), and lymphocyte accumulation (77.8%).

**Conclusions:**

PDX model of PMCA‐S was established successfully, and i.p. 5‐FU could inhibit tumor proliferation and progression, with decreased Ki67 positive rate and ePCI score. Hepatotoxicity was the main side effect.

## Graphical abstract

In the study, we successfully established patient‐derived xenograft model of pseudomyxoma peritonei, and proved that 5‐fluorouracil administrated intraperitoneally is effective in pseudomyxoma peritonei.

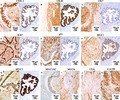

## 1 INTRODUCTION

Pseudomyxoma peritonei (PMP) is a malignant condition characterized by mucinous ascites, widespread intraperitoneal dissemination and omental cake,^1^, ^2^ with incidence rate being around 1‐2/million.^3^ PMP mainly results from the perforation of appendiceal mucinous tumor, following “redistribution phenomenon” of free tumor cells and mucus in the abdominal cavity. Peritoneal Surface Oncology Group International (PSOGI) classified PMP into four categories according to the microscopic manifestations^1^: acellular mucin (AC), low‐grade mucinous carcinoma peritonei (or disseminated peritoneal adenomucinosis, DPAM), high‐grade mucinous carcinoma peritonei (or peritoneal mucinous carcinomatosis, PMCA), high‐grade mucinous carcinoma peritonei with signet ring cells (or peritoneal mucinous carcinomatosis with signet ring cells, PMCA‐S).

PMP usually presents as an inertial and chronic course. After traditional treatment, frequent relapses occur and repeated surgeries are required to relieve the symptom of bowel obstruction. The 5‐year and 10‐year survival rates are 53%‐75% and 10%‐32%,^4^, ^5^ respectively. In 2014, PSOGI recommended cytoreductive surgery (CRS) and hyperthermic intraperitoneal chemotherapy (HIPEC) as the standard treatment for PMP. Clinical studies have proved that optimal CRS + HIPEC did improve overall survival (OS) up to 196 months.^6^

Currently, PMP is considered as a loco‐regional malignancy, with few distant metastases.^7^ It is generally believed that systemic chemotherapy, whether used singly or combined with other drugs, is not effective.^8^ Due to the existence of “peritoneum‐plasma barrier,” drugs administrated by intraperitoneal (i.p.) injection are relatively limited to the peritoneal cavity, leading to higher drug concentration than blood vessel. Sugarbaker et al^9^ performed early postoperative intraperitoneal chemotherapy (EPIC) on the basis of CRS + HIPEC. Huang et al^10^ proved that EPIC is an independent prognostic factor for better survival. However, there lacks laboratory evidence recommending i.p. administration of 5‐fluorouracil (5‐FU). Our study aims to establish PMP patient‐derived xenograft (PDX) model, and evaluate the efficacy and toxicity of i.p. injection of 5‐FU.

## 2 MATERIALS AND METHODS

### 2.1 Patient and tumor sample

Tumor sample was obtained from a 61‐year‐old female patient with PMP history for 8 years. She received four times of CRS and was diagnosed with PMCA originating from appendix. Tumors from the last surgery was collected for grafting, with informed consent from the patient. This study had been approved by Scientific Research Ethics Committee of Beijing Shijitan Hospital, Capital Medical University [Approval number: 2018 Research Ethics Review No. (73)], and was performed under the guideline of the Declaration of Helsinki.

### 2.2 Establishment of PMP PDX model

#### 2.2.1 Animals

Specific pathogen free BALB/c nu/nu mice, 4‐5 weeks old, 13‐15 g, were from Beijing HFK Bio‐Technology (Beijing, China; animal quality certificate No. SCXK (Jing) 2014‐0004) and maintained in individually ventilated cages in a barrier environment at Beijing Percans Oncology Co. Ltd. (Beijing, China; animal quality certificate No. SYXK (Jing) 2015‐0030). The animals were acclimatized for 1 week before experiment. All procedures were approved by Scientific Research Ethics Committee of Beijing Shijitan Hospital, Capital Medical University [Approval number: 2018 Research Ethics Review No. (73)].

#### 2.2.2 Model construction

The tumor specimens were cleaned with RPMI 1640 medium (Corning, New York, USA, catalog number 10‐040‐CVR), cut into 3‐5 mm^3^ pieces and then aspirated into a 25G trocar sheath needle, which was used for the blunt separation of connective tissue, following subcutaneous (s.c.) injection in the roots of 4 limbs through the push of the inner core needle. Subcutaneous tumor was measured for volume weekly and resected for passaging as it reached 500 mm^3^. Part of the s.c. tumor was frozen for future passage. Firstly, tumor sample was cut into 3 × 3 × 3 mm^3^ pieces and immersed in adequate conservation medium with 60% RPMI 1640 medium, 30% fetal bovine serum (Hangzhou Sijiqing Bioengineering Materials, Hangzhou, China, catalog number 11011‐b615) and 10% dimethyl sulfoxide (Ameresco, Fremingham, USA, catalog number 0231‐500 mL). Then tumor was placed in Nalgene programmed cooling box (New York, USA, catalog number 5100‐0001) at −80°C overnight and finally stored in liquid nitrogen. Upon resuscitation, frozen tumor was incubated at 37°C until complete melting. Subcutaneous models were reestablished following the methods above.

After six stable passages, s.c. tumor was obtained and homogenized on ice with RPMI 1640 medium to make tumor cell suspension. Ten female mice were anesthetized by sodium pentobarbital, then 60 μL tumor cell suspension per mouse was injected through a 0.5‐1.0 cm incision in the mid‐upper abdominal wall, into in both sides of upper and lower abdominal quadrant and the flank. The incision was closed with 4‐0 silk sutures. General status of mice was monitored daily, and the body weight and abdominal girth were measured every 2 to 3 days.

#### 2.2.3 Intraperitoneal injection of 5‐FU to treat PMP PDX model

**Figure 1:**
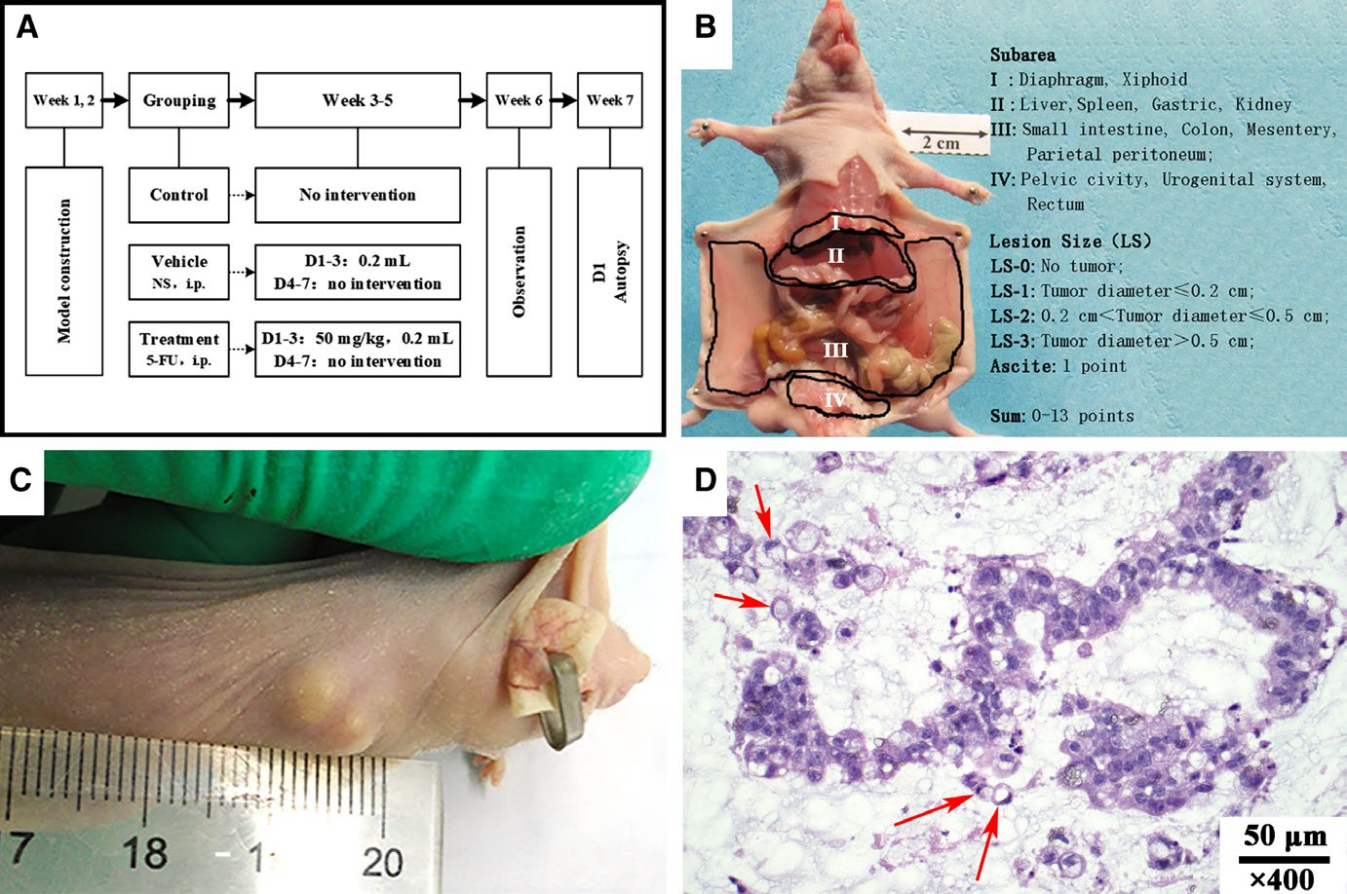
Experimental procedures, experimental peritoneal cancer index (ePCI), and the construction of subcutaneous model. A, Experimental procedures; B, The subarea and scoring of ePCI score system; C, Globular subcutaneous tumor; D, Histopathology showed peritoneal mucinous carcinomatosis with signet ring cells. Red arrow points to signet ring cells

Two weeks after grafting, 30 mice (half male, half female) were randomized into three groups: control, vehicle, and treatment groups (n = 10 for each group). Treatment lasted for 3 weeks. Control group was left free without any intervention. Vehicle and treatment group were i.p. injected with normal saline (0.2 mL) and 5‐FU (50 mg/kg, 0.2 mL, Shanghai Xudong Haipu Pharmaceutical Co., Ltd., catalog number H31020593), respectively, 1 time per day from days 1 to 3 (D1‐3) of each week, leaving D4‐7 for recovery (Figure 1A).

#### 2.2.4 Gross pathological study

One week after treatment period, mice were sacrificed for autopsy. Tumor growth and progression features were recorded: sites of dissemination, tumor volume, mucinous ascites, peritoneal implantation, and organ infiltration. Experimental peritoneal cancer index (ePCI) were used to evaluate the extent of tumor dissemination, based on the published studies by Shao^11^ and Steller.^12^ The abdominal‐pelvic cavity was divided into four subareas (Figure 1B), and lesion size score (LS) in each subarea is determined by the diameter of the largest tumor: LS‐0, no visible tumor; LS‐1, diameter ≤ 0.2 cm; LS‐2, 0.2 cm < diameter ≤0.5 cm；LS‐3, diameter > 0.5 cm; and Mucinous ascites, 1 point. The accumulative ePCI score ranges from 0 to 13. ePCI score was evaluated and checked by operator and recorder at the same time.

#### 2.2.5 Hematoxylin‐eosin (H&E) staining and immunohistochemistry (IHC) analysis

Three models were randomly selected from each group for H&E and IHC analysis. Tumors were fixed in 10% neutral buffered formalin for 48 hours, following routine dehydration, paraffin embedding and section (4‐6 μm). H&E staining (Dako Hematoxylin, Dako Eosin and Dako Bluing Buffer, catalog number CS701) was performed on Dako CoverStainer for H&E (Agilent Technologies, Inc). Sections for IHC analyses were baked for 1 hour, deparaffinized in dimethylbenzene for two times, rehydrated through a graded series of alcohol to distilled water, following antigen retrieval by heat treatment for 4 minutes in citrate buffer (pH 8.4) and quenching of endogenous peroxidase activity for 10 minutes using 3% hydrogen peroxide. Antibodies and horseradish peroxidase were incubated for 40 and 25 minutes at room temperature, respectively. The specimens were performed on intelliPATH FLX (BIOCARE MEDICAL, LLC) with Polymer Immunohistochemical Detection System **(**Wuxi OriGene Technologies, Inc, catalog number MA‐2000). Antibodies came from OriGene, including: mucin1 (MUC1, clone MRQ‐17, catalog number ZM‐0391), mucin2 (MUC2, clone MRQ‐18, catalog number ZM‐0392), mucin5AC (MUC5AC, clone MRQ‐19, catalog number ZM‐0395), mucin6 (MUC6, clone MRQ‐20, catalog number ZM‐0396), carcinoembryonic antigen (CEA, clone 12‐140‐10, catalog number ZM‐0062), carbohydrate atigen199 (CA199, clone C241: 5: 1: 4, catalog number ZM‐0021), cytokeratin7 (CK7, clone UMAB161, catalog number ZM‐0071), cytokeratin20 (CK20, clone EP23, catalog number ZA‐0574), VILLIN (clone EP163, catalog number ZA‐0575), caudal‐type homeobox transcription factor‐2 (CDX‐2, clone EP25, catalog number ZA‐0520). Staining of antibodies protein53 (p53, clone DO7, catalog number ZM‐0408) and Ki67 (clone UMAB107, catalog number ZM‐0166) were performed on LEICA BOND‐III (Leica Biosystems) with Bond™ PolymerRefine Detection (Leica Biosystems, catalog number DS9800). Pictures were taken by Zeiss Axio Scope.A1 (Carl Zeiss AG) and Mshot Microscopic Camera MS60 (Guangzhou Mshot Photoelectric Technology Co., Ltd.). Ki67 positive rate was analyzed with Image Pro Plus V6.0 (Media Cybernetics).

For toxicity evaluation, heart, lung, liver, spleen, and kidney were resected and fixed in 10% neutral buffered formalin for 48 hours. H&E staining was performed following methods above. All H&E and IHC sections were reviewed by the author and two experienced senior pathologists. By comparing with control group, any histopathological changes were considered toxic reaction.

#### 2.2.6 Whole‐exome sequencing (WES)

We followed the WES protocol developed by Giannakis et al,^13^ with minor modifications. Briefly, for model tumor study, 2 liquid nitrogen‐preserved tumor tissues from seventh generation were homogenized, centrifuged at 9400 g for 1 minutes, and the supernatant was discarded. For the patient tumor study, 1 formalin‐fixed, paraffin‐embedded (FFPE) tumor sample from colonic metastasis was used for sequencing, with 1 colon mucosa sample as normal control. Genomic DNA was extracted using QIAGEN DNA kit (QIAGEN NV, Hilden, Germany, catalog number 180 134), and the DNA quality was checked using Quant‐iT Pico Green dsDNA Assay Kit (Invitrogen/ThermoFisher SCIENTIFIC, Massachusetts, USA, catalog number P11496). One microgram of qualified DNA was obtained to construct whole‐exome capture libraries (final concentration > 20 ng/μL) after shearing, end repair, phosphorylation and ligation to barcoded sequencing adaptors. DNA was captured using SureSelectXT Human All Exon V6 (Agilent Technologies, California, USA, catalog number 5190‐8864) and was then sequenced on the Illumina X10 platform (Illumina Inc, California, USA), with average sequencing coverage being 100×. WES data underwent mutation analysis using human genome build hg19 as the reference genome. Somatic SNVs was analyzed via MuTect algorithm^14^ and somatic indels was detected with both Indelocator (http://www.broadinstitute.org/cancer/cga/indelocator) and Strelka algorithms (V2.9.10).^15^ The sequenced reads were realigned to the hg19 by Burrows‐Wheeler Aligner BWA‐MEM (V0.7.8, http://biobwa.sourceforge.net/) to enhance valid SNVs. The original sequencing datasets have been submitted to NCBI (Accession numbers: SRR10028120, SRR10028121, SRR10028122, and SRR10028123).

#### 2.2.7 Statistical analysis

All statistical analyses were performed on SPSS V24.0 (IBM SPSS Statistics). The body weights, ePCI scores, and Ki67 positive rates were expressed as mean ± standard deviation. In model construction study, the difference of ePCI scores between D30 group and D47 group were analyzed with two‐tailed Mann‐Whitney U test. In efficacy and toxicity studies, the comparison of body weights and Ki67 positive rates among control, vehicle, and treatment group were analyzed with Repeated Measurement Two‐way ANOVA and One‐way ANOVA respectively. Bonferroni test was used for post hoc multiple comparisons. The comparison of ePCI scores among three groups was analyzed by Kruskal‐Wallis test. *P* < .05 was considered statistically significant.

## 3 RESULTS

### 3.1 Subcutaneous PMP model

The tumor specimens were s.c. injected into 3 mice and one developed subcutaneous tumor. The tumor reached 100 mm^3^ on day 51, and formed typical nodule (Figure 1C). The average passage interval was 44.2 ± 5.2 days. Microscopically, tumor tissue from resuscitated model was diagnosed with PMCA‐S (Figure 1D). This subcutaneous tumor from the sixth passage was used to establish orthotopic PMP model.

### 3.2 Orthotopic PMP model

#### 3.2.1 General status

**Figure 2:**
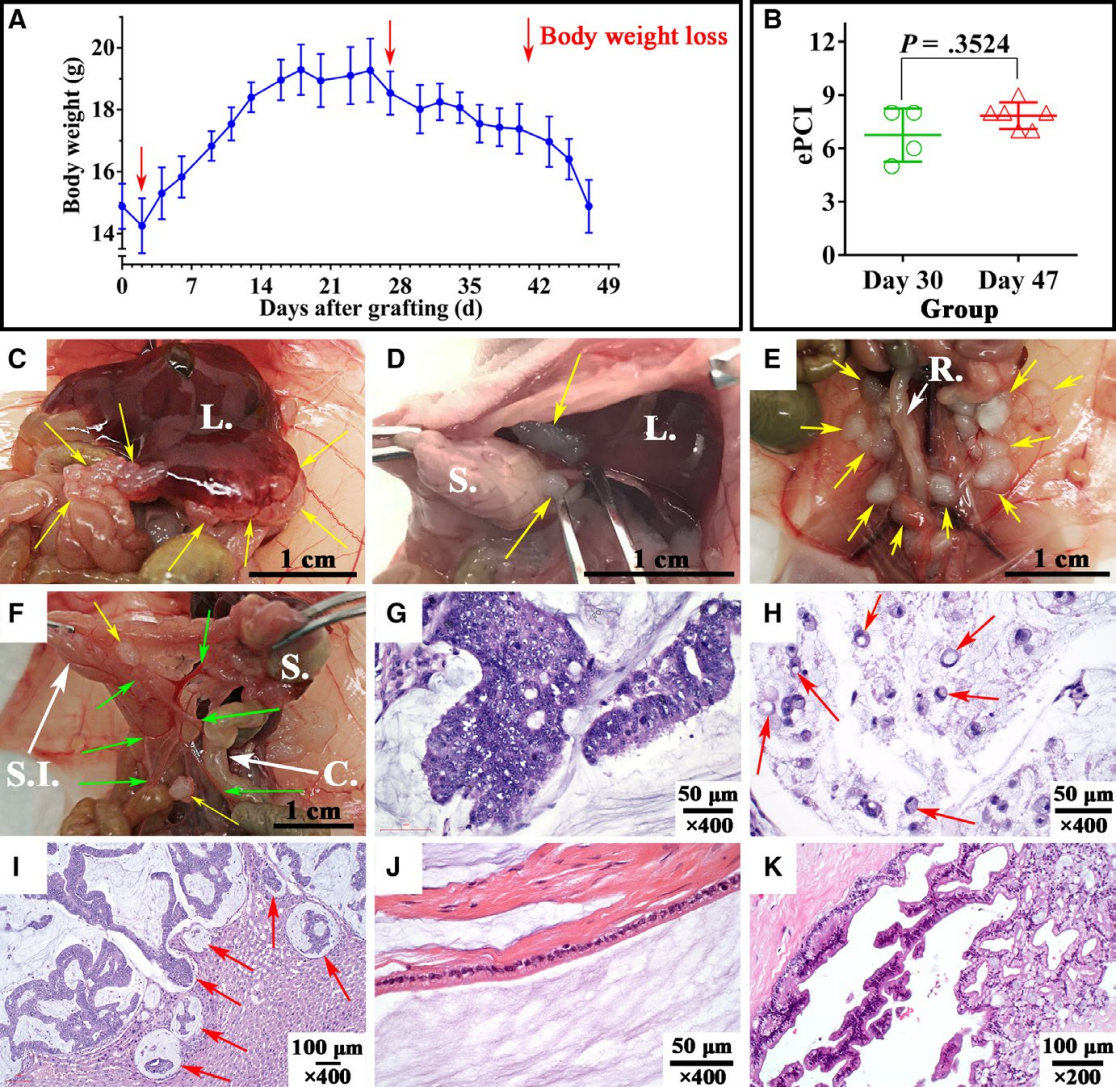
Body weight changes, gross pathology of 10 models and histopathology of models and patient. A, Body weight changes of 10 models; B, Experimental peritoneal cancer index score of D30 group and D47 group, analyzed by two‐tailed Mann‐Whitney U test; C, Giant tumor nodule was tightly adhered to liver; D, Tumor nodule between liver and stomach and tumor on the surface of greater curvature; E, Multiple tumor nodules in retroperitoneum; F, Mesenteric thickening, adhesion and contracture (green arrow), and mesenteric tumor (yellow arrow); G, Microscopic features of models. Tumor tissue consisted of multiple layers of tumor cells. Hyperchromasia, mitotic figures were observed (×400); H, Signet ring cells (red arrow, ×400); I, Liver lobule was infiltrated by mucus pools with floating tumor tissue (red arrow); J&K, Microscopic picture of patient tumor tissue showed partly DPAM (J, ×400), and partly PMCA (K, ×200). L., liver; S., stomach; R., rectum; SI, small intestine; C., colon. Hematoxylin‐eosin staining was repeated for 3 times for model histopathological identification

From the day of grafting to autopsy, the activity, mental state, diet, stool and urine of mice showed normal. On D2 after grafting, body weight of mice dropped slightly, followed by a stable weight gain. Since D27, body weight decreased remarkably, being 20.7% lower on D47 than D25 (Figure 2A). No adverse event occurred during model construction.

#### 3.2.2 Gross pathological presentation

The successful rate of model construction was 100% (10/10). Four mice were sacrificed on D30, with the other 6 autopsied on D47. No obvious abdominal distension was observed. The models were characterized by a variable number of semi‐solid mucinous tumor of different sizes. Most of the mucinous tumor were loosely adhered to abdominal organ/areas, including: xiphoid area, 20% (2/10); diaphragm, 10% (1/10); liver, 50% (5/10); spleen, 30% (3/10); stomach, 30% (3/10); kidney, 20% (2/10); peritoneal surface, 100% (10/10); mesentery, 60% (6/10); and pelvic cavity, 90% (9/10).

All the 4 subareas were involved by tumor in both D30 and D47 group. However, mice in D47 group had more tumor nodules, larger tumor volume, and wider dissemination, with ePCI score being 7.8 ± 1.0 and 6.8 ± 1.5 (*P* = .3524, Figure 2B), respectively. Mesenteric thickening, adhesion and contracture were newly found on D47 group compared with D30 group (Figure 2C‐F).

#### 3.2.3 Histopathology and immunohistochemistry

The orthotopic model was again confirmed as PMCA‐S (Figure 2G,H): Multiple mucus pools with poorly differentiated mucinous epithelium and even signet ring cells (Figure 2H) inside were separated by fibrous band. Tumor tissue consisted of multiple layers of tumor cells, presenting cable, cluster, and adenoid structure. Hyperchromasia and mitotic figures were frequently observed, indicating significant atypia. Moreover, liver infiltration (Figure 2I) was found in the model. Compared with the histopathological diagnosis of partly DPAM (Figure 2J) and partly PMCA (Figure 2K) in patient, this model presented higher degree of malignancy.

**Figure 3:**
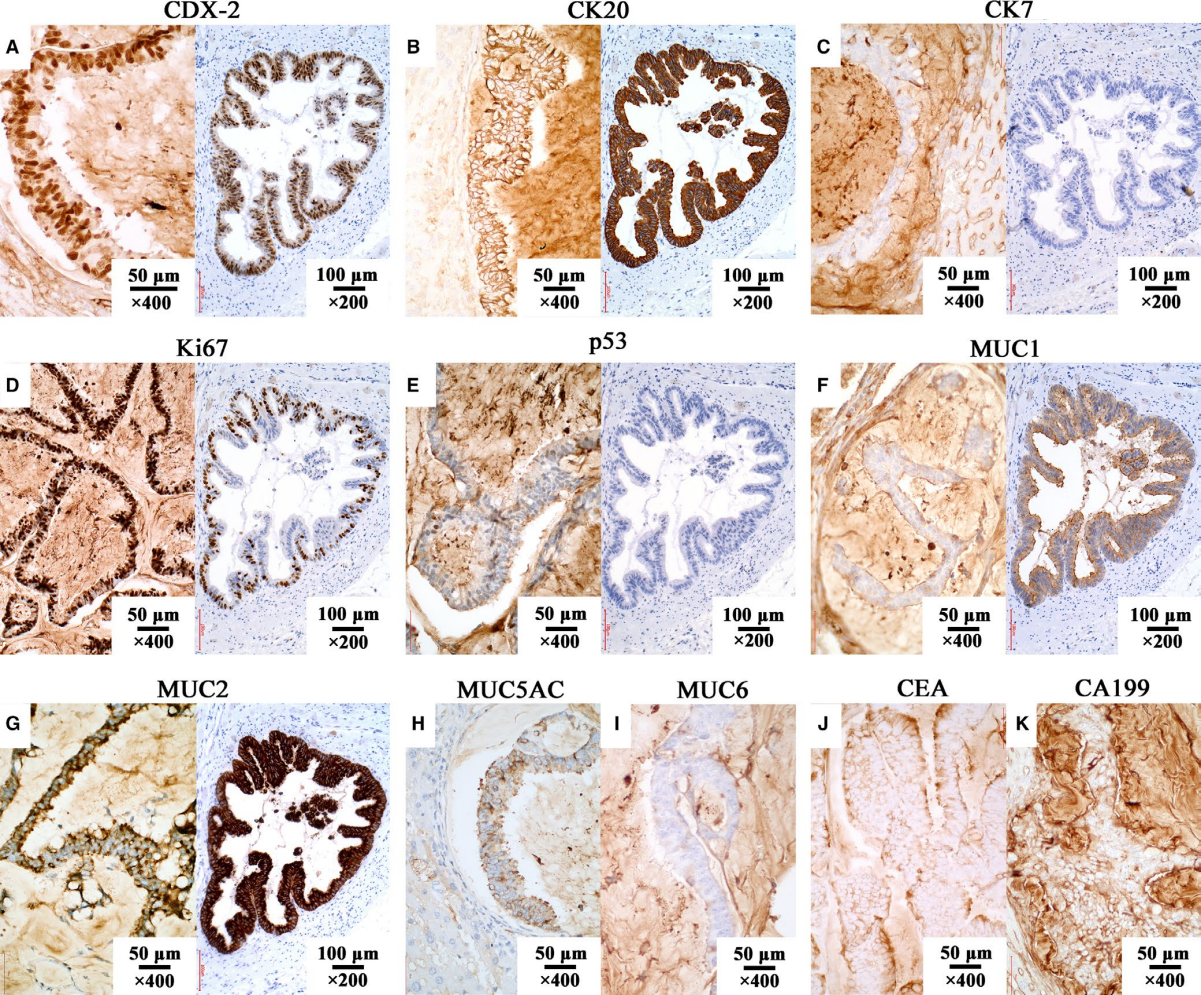
Immunohistochemistry of model and patient (Figure A‐G: left image, model; right image, patient; Figure H to K, model). A: CDX‐2 +; B: CK20 +; C: CK7 ‐; D: Left image, Ki67 + (80%‐90%); right image, Ki67 + (25%‐50%); E: p53 ‐; F: MUC1 +; G: MUC2 +; H: MUC5AC +; I: MUC6 ‐; J: CEA +; K: CA199 +; The magnifications of model and patient are × 400 and × 200 respectively. Immunohistochemistry analysis were repeated for 3 times

Immunohistochemistry study showed positive for MUC1, MUC2, MUC5AC, CEA, CA199, CK20, CDX‐2, VILLIN, and Ki67 (+80%‐90%), and negative for MUC6, CK7, p53 (Figure 3). Except for higher Ki67 positive rate (80%‐90% vs 25%‐50%, Figure 3D), the immunohistochemical presentation of PDX model was identical with patient.

#### 3.2.4 Gene mutation

*KIT*, *APC*, *GNAS*, and *PIK3CA* mutation was found in PMP PDX model, among which the most significant gene mutation is the missense mutation in exon 10 (c.1621A>C) of *KIT* gene (Table 1). Twenty‐eight gene mutations were found in patient, including typical *KRAS* and *GNAS* mutations in PMP (Table 1). The models and the patient shared 1 common mutation, *GNAS* mutation, with both mutation site located in chromosome 20:57 484 421 (calling G > A).

**Table 1:** Gene mutation of PMP PDX model and patient

	Gene	Exon	Base mutation	Amino acids mutation	Mutation abundance^a^	Type of mutation
Model	*KIT*	9	c.1481A > C	p.Y494S	6.1%	M
		9	c.1499C > G	p.T500S	6.2%	M
		10	c.1621A > C	p.M541L	89.7%	M
		14	c.2088T > G	p.D696E	27.9%	M
		14	c.2091T > G	p.H697Q	28.3%	M
		14	c.2129A > C	p.K710T	32.7%	M
		14	c.2134T > C	p.S712P	32.6%	M
	*GNAS*	8	c.2531G > A	p.R844H	45.7%	M
	*PIK3CA*	2	c.112C > T	p.R38C	40.4%	M
	*APC*	10	c.847C > T	p.R283X	40.7%	N
		17	c.4012C > T	p.Q1338X	40.6%	N
Patient	*CACNA1E*	37	c.5050G > A	p.A1684T	20.4%	M
	*KRAS*	2	c.35G > T	p.G12V	11.7%	M
	*SEPT9*	2	c.453G > T	p.Q151H	10.0%	M
	*KDM5C*	4	c.463T > C	p.S155P	9.4%	M
	*CTNND2*	12	c.2137C > T	p.R713C	9.3%	M
	*ABCD1*	1	c.475G > A	p.A159T	8.9%	M
	*POU4F1*	2	c.1075C > T	p.R359W	8.7%	M
	*ZNF653*	5	c.1330A > T	p.K444X	8.4%	S
	*CHAC1*	3	c.566C > T	p.P189L	8.3%	M
	*SCAPER*	2	c.77G > A	p.R26K	7.8%	M
	*ZNF667*	5	c.1714C > T	p.R572X	7.7%	S
	*SETMAR*	3	c.1514G > A	p.R505Q	7.2%	M
	*SUPT20H*	12	c.908T > G	p.I303R	7.2%	M
	*RFC3*	3	c.245T > A	p.I82N	6.8%	M
	*ZFP37*	1	c.118G > T	p.E40X	6.7%	S
	*PRH2*	3	c.124A > C	p.I42L	6.6%	M
	*SGCG*	4	c.320C > A	p.S107X	6.3%	S
	*TTLL5*	6	c.388C > T	p.R130W	6.1%	M
	*GNAS*	8	c.602G > A	p.R201H	5.9%	M
	*KMT2C*	14	c.2263C > T	p.Q755X	5.3%	S
	*KRTAP23‐1*	1	c.37C > T	p.H13Y	5.3%	M
	*SPDYE1*	5	c.700G > A	p.G234R	5.1%	M
	*KRT84*	5	c.977G > A	p.R326H	5.0%	M
	*ARHGAP44*	16	c.1387G > A	p.V463M	4.5%	M
	*KCNK13*	2	c.580G > A	p.V194M	4.3%	M
	*ZNF644*	4	c.3386G > A	p.R1129H	4.1%	M
	*WNT5A*	5	c.869G > A	p.R290H	4.0%	M
	*MUC17*	3	c.8868G > C	p.R2956S	2.7%	M

Abbreviations: M, missense mutation; N, nonsense mutation; S, stop gain mutation.

a Mutation abundance = Mutation type/(Mutation type + Wild type) × 100%.

### 3.3 Efficacy and toxicity study of i.p. injection of 5‐FU in PMP model

#### 3.3.1 General status of the models

**Figure 4:**
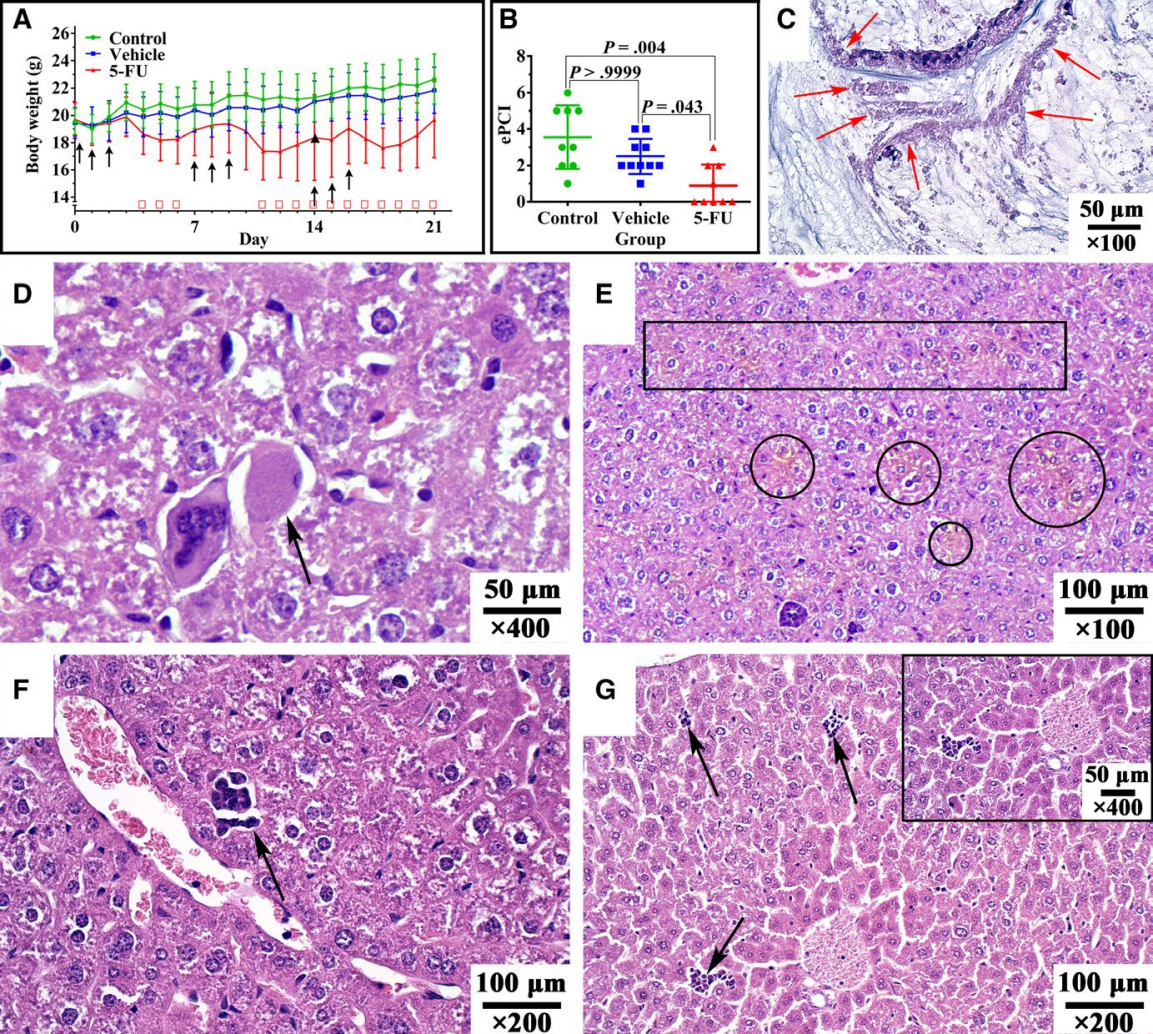
Body weight changes, experimental peritoneal cancer index (ePCI) changes, histopathology changes and hepatotoxicity after 5‐fluorouracil (5‐FU) treatment. A, Body weight changes of 30 mice. ↑: normal saline (vehicle group) or 5‐FU (treatment group) was intraperitoneally injected; ▲: one mouse in treatment group died on day 14; ※: differences of body weight among the 3 groups were statistically significant; B, EPCI score of 5‐FU groups is lower than the other 2 groups; Comparison of body weight and ePCI score were analyzed by Repeated Measurement Two‐way ANOVA and Kruskal‐Wallis test respectively; C: Homogeneous eosinophilic cell fragment floating in mucus pool after intraperitoneal injection of 5‐FU, remaining the shape of striped epithelium (×400); D: Arrow pointed acidophilic body (×400); E: Circle/square showed areas of cholestasis (×100); F: Arrow showed bile canaliculus hyperplasia and obstruction (×200); G: Arrows showed lymphocytes accumulation (×200; top right: ×400). Hematoxylin‐eosin staining was repeated for 3 times for efficacy and toxicity evaluation

During 2 weeks’ model construction, the general status of 30 mice were normal, with stable body weight gain. On D0, body weight differences among 3 groups had no statistical significance (*P* = .868, Figure 4A). The gross pathology, histopathology, and immunohistochemistry presentations were identical to former generation.

#### 3.3.2 Intraperitoneal injection of 5‐FU lowered ePCI score

The comparison of ePCI score among 3 groups are shown in Figure 4B. The ePCI score of control, vehicle, and treatment groups are 3.6 ± 1.7, 2.7 ± 1.1, and 0.9 ± 1.2, respectively. Kruskal‐Wallis test showed that differences among the 3 groups was statistically significant (*P* = .004 for all; *P* = .004, treatment group vs control group; *P* = .043, treatment group vs vehicle group; *P* > .9999, control group vs vehicle group).

#### 3.3.3 Histopathological changes of tumor tissue after 5‐FU treatment

The histopathological presentation of tumor tissue from control group and vehicle group remained PMCA‐S. While necrosis was observed microscopically, with homogeneous eosinophilic cell fragment floating in mucus pool after 5‐FU treatment, and remaining the shape of striped epithelium (Figure 4C). Ki67 positive rate of treatment group was 57.3% ± 15.1%, lower than control group (72.2% ± 15.1%) and vehicle group (70.0% ± 14.0%) (*P* = .010 for all; *P* = .028 for treatment group vs control group; *P* = .032 for treatment group vs vehicle group; *P* > .9999 for control group vs vehicle group).

#### 3.3.4 Toxicity evaluation of 5‐FU: Influence on body weight

During treatment period, 5‐FU had significant negative impact on body weight (*P* = .010, Repeated Measurement Two‐way ANOVA) (Figure 4A). The body weight of treatment group was significantly lower than the other 2 groups on D4, D5, and D6, and were able to recover on D7 to D10 after treatment ceases. However, body weight fluctuated from D11 to D21, with 1 mouse died on D14. Body weight of the dead mouse was 17.9 g, 8.2% lower than the average body weight. At the end of the treatment (D21), the body weight of control, vehicle, and treatment groups were 22.7 ± 1.8 g, 21.9 ± 1.7 g, and 19.7 ± 2.8 g (*P* = .019 for all; *P* = .021, treatment group vs control group; *P* = .111, treatment group vs vehicle group; *P* > .9999, control group vs vehicle group). Control and vehicle groups had similar and stable weight gain during treatment.

#### 3.3.5 Organ toxicity

Gross pathology revealed congestive splenomegaly (8/9, 88.9%), and no abnormal change was found in heart, lung, liver, and kidney. Microscopically, organ toxicity was mainly found in liver (9/9, 100%), including: acidophilic body (5/9, 55.6%, Figure 4D), cholestasis (9/9, 100%, Figure 4E), bile canaliculus hyperplasia and obstruction (2/9, 22.2%, Figure 4F), lymphocyte accumulation (7/9, 77.8%, Figure 4G). No liver toxicity was found in control group and vehicle group.

## 4 DISCUSSION

Using tumor specimens from surgery, we established PMP PDX model replicating human PMP, in terms of gross pathology, histopathology, and immunohistochemistry. Hence the model was thought to be suitable for drug efficacy and toxicity evaluations. The result revealed that 5‐FU (50 mg/kg, i.p.) reduced tumor burden in abdominal cavity, inhibited tumor dissemination, and lowered ePCI score. Histopathology revealed that 5‐FU resulted in tumor necrosis and lowered Ki67 positive rate. Therefore, i.p. injection of 5‐FU is a feasible treatment for PMP.

In model establishment study, body weight of mice decreased slightly 2 days after operation, implying that mice are sensitive to interventional operation and objectively reflect the general status. When the body weight decreased dramatically again on D27, it was considered that model had entered clinically advanced stage. In animal study, body weight could be an ideal indicator for estimating tumor growth or stage. Therefore, treatment should be applied before this stage to get better effect.

Autopsy revealed widespread tumor dissemination, involving most of the organs in the abdominal‐pelvic cavity, which replicated advanced‐stage PMP patient. Although the ePCI score on D47 was higher than D30, there was no statistical significance (*P* = .3524), suggesting the inertial and chronic course of PMP.

There are already several kinds of PMP PDX models worldwide, covering DPAM,^16^, ^17^ PMCA,^17^, ^18^, ^19^ and PMCA‐S.^20^ All of them replicated human PMP, with typical abdominal distention, mucinous tumor lesions without parenchymal invasion, gelatinous ascites, similar pathological and immunohistochemical findings. Our study had constructed a PMP model with similar characteristics, but several differences need to be discussed. First, this is the first model showing parenchymal invasion, which was in accordance with the patient. However, the model got higher degree of malignancy than patient microscopically, as has been reported by previous study.^18^ It is possible that the biological behavior of tumor evolves faster in immune‐deficient animals, and fully reflects the malignant characteristics of tumor, such as proliferation, invasion and heterogeneity. Second, despite of multiple mucus pools observed microscopically, our model lacked a large volume of gelatinous ascites and abdominal distension, which is common in previous studies. We deduced that several passages of subcutaneous model may had caused this phenomenon by influencing tumor microenvironment and the secretion of mucus. However, considering abundant mucus pools observed microscopically and other parameters being similar to the patient, it is a suitable PMP model for drug study (chemotherapy drugs or mucolytic drugs).

Whole‐exome sequencing revealed that the model and patient shared *GNAS* mutation, which was a frequent mutation in PMP^21^, ^22^ and was thought to be related to mucin hypersecretion.^23^ Considering the same mutation site in chromosome 20:57484421, we thought that the characteristic of *GNAS* mutation was preserved during the construction and passaging of PMP model. However, *KRAS* mutation and other mutations in the patient were missed in the model by whole‐exome sequencing, with newly detected mutations in *KIT*, *APC*, and *PIK3CA* genes. One of the possible reasons causing different mutation profiles between patient and model is the different tumor sampling sites during model construction and FFPE section. Besides, mutations status drifting during model passaging might also contribute to the variances. Comparing the high mutation abundance in models and low mutation abundance in patients (Table 1), we could infer that the tumor sampling sites used for grafting may have caused this difference. Although changes might occur to the mutation profiles in murine model, few papers on PMP model have reported the similarities or differences between mutation profiles of PDX model and patient.^17^ Despite the similarities between gross pathology, histopathology, and IHC, the model may only partly reflect the molecular characteristic of PMP, which however determined the promising function in gene and molecular experiments. In terms of the mutation profiles, we could say that this model is promising for *GNAS* mutation studies.

One of the most important functions of this models is to provide experimental platform for interventional study. The ePCI score for control group ranges from 1‐6 points (total score: 0‐13 points) 5 weeks after tumor grafting, indicating that the time of treatment located in the early to median stage of PMP. Our result proved that i.p. injection of 5‐FU in the early to median stages did inhibit tumor progression compared with control and vehicle group. Some mice were even cured, with ePCI of 5 mice in the treatment group being 0. The result has provided laboratory evidence for clinical application of i.p. 5‐FU.

Although the concentration of 5‐FU in blood is significantly lower than abdominal cavity due to “peritoneum‐plasma barrier,” i.p. 5‐FU still causes organ toxicity and even death. Hepatotoxicity was the most severely impacted organ among the 5 organs studied. The way 5‐FU is metabolized may explain this phenomenon: it's mainly metabolized through liver and thus enhanced oxidative stress.^24^ Therefore, during clinical application of i.p. 5‐FU, liver function should be closely monitored and liver protection therapies should be emphasized.

PMP is characterized by infiltration on the surface of abdominal and pelvic peritoneum, and systemic chemotherapy hardly works. The ratio of areas under curve of i.p./iv injection reach as high as 117 due to special pharmacokinetics of i.p. 5‐FU.^25^ Through i.p. administration, drug concentration in the abdominal cavity is much higher than blood vessel, which contributed to better tumor‐killing effect and reduced systemic toxicity at the same time. However, nephrotoxicity, and cardiotoxicity had been reported in non‐PMP animal studies,^26^, ^27^ as well as case report on sudden cardiac death after i.p. administration of 5‐FU.^28^ Besides, though i.p. injection of 5‐FU had been applied in clinical treatment of PMP,^29^, ^30^, ^31^ the efficacy is unclear because of the influence of large volumes of mucus. Currently, treatment efficacy evaluation of i.p. 5‐FU was limited to animal models of colorectal cancer.^32^, ^33^, ^34^ Compared with these studies, our study established a typical PMP PDX model, taking into consideration of both drug efficacy and toxicity assessment, which is helpful to comprehensively evaluate the efficacy and side effects of 5‐FU.

Our study has limitations. Mucinous ascites and abdominal distention were not obvious in the model. Besides, there was no direct gross‐pathological and histopathological data of the dead 1 mouse in treatment group.

In summary, this PDX model of PMCA‐S is an optimal platform for both drug evaluation and mechanism study of PMP. Intraperitoneal injection of 5‐FU could inhibit tumor proliferation and progression, decreasing Ki67 positive rate and ePCI score. Our study provides experimental evidence for the clinical application of i.p. 5‐FU.

## 5 AUTHOR CONTRIBUTIONS STATEMENT

Yan Li contributed to study concepts. Yan Li and Yu‐Lin Lin contributed to study design. Yu‐Lin Lin contributed to literature research. Yu‐Lin Lin and Qian Chen contributed to experimental studies. Yu‐Lin Lin, Jue Zhang, Feng‐Cai Yan, Xi Jiang, Ru Ma, and Zhi‐Ran Yang contributed to data acquisition. Yu‐Lin Lin, and Yan Li contributed to data analysis/interpretation. Yu‐Lin Lin contributed to statistical analysis. Yu‐Lin Lin contributed to manuscript preparation. Yan Li contributed to manuscript definition of intellectual content. Yu‐Lin Lin and Yan Li contributed to manuscript editing. Yan Li, Hongbin Xu, and Zheng Peng contributed to manuscript revision/review. Yan Li contributed to manuscript approval.

## CONFLICT OF INTEREST

The authors declare that they have no competing interests.

## Data Availability

The data that support the findings of this study are openly available in [repository name e.g “figshare”] at http://doi.org/%5Bdoi], reference number [reference number].
